# Risk of bias assessment of sequence generation: a study of 100 systematic reviews of trials

**DOI:** 10.1186/s13643-018-0924-1

**Published:** 2019-01-08

**Authors:** Francesca Wuytack, Maria Regan, Linda Biesty, Pauline Meskell, Jennifer E. Lutomski, Martin O’Donnell, Shaun Treweek, Declan Devane

**Affiliations:** 1 School of Nursing & Midwifery, Trinity College Dublin, 24 D’Olier Street, Dublin 2, Ireland; 2 School of Medicine, National University of Ireland Galway, Galway, Ireland; 3 HRB-Trials Methodology Research Network/School of Nursing & Midwifery, National University of Ireland Galway, Galway, Ireland; 4 Department of Nursing & Midwifery, Health Sciences Building, University of Limerick, Limerick, Ireland; 5 Radboud Biobank, Radboud University Medical Center, Geert Grooteplein 10, 6525 GA Nijmegen, The Netherlands; 6 Health Services Research Unit, University of Aberdeen, Health Sciences Building, Foresterhill, Aberdeen, AB25 2ZD UK

**Keywords:** Randomisation, Sequence generation, Systematic reviews, Risk of bias, Quality of evidence

## Abstract

**Background:**

Systematic reviews of randomised trials guide policy and healthcare decisions. Yet, we observed that some reviews judge randomised trials as high or unclear risk of bias (ROB) for sequence generation, potentially introducing bias. However, to date, the extent of this issue has not been well examined. We evaluated the consistency in the ROB assessment for sequence generation of randomised trials in Cochrane and non-Cochrane reviews, and explored the reviewers’ judgement of the quality of evidence for the related outcomes.

**Methods:**

Cochrane intervention reviews (01/01/2017–31/03/2017) were retrieved from the Cochrane Database of Systematic Reviews. We also searched for systematic reviews in ten general medical journals with highest impact factors (01/01/2016–31/03/2017). We examined the proportion of reviews that rated the sequence generation domain as high, low or unclear risk of selection bias. For reviews that had rated any randomised trials as high or unclear risk of bias, we examined the proportion that had assessed the quality of evidence.

**Results:**

Overall, 100 systematic reviews were included in our analysis. We evaluated 64 Cochrane reviews which comprised of 984 randomised trials; 0.8% (*n* = 8) and 52.2% (*n* = 514) were rated as high and unclear ROB for sequence generation respectively. We further evaluated 36 non-Cochrane reviews which comprised of 1376 trials; 5.8% (*n* = 80) and 39.6% (*n* = 545) were rated as high and unclear ROB respectively. Ninety percent (*n* = 10) of non-Cochrane reviews which rated randomised trials as high ROB for sequence generation did not report an underlying reason. All Cochrane reviews assessed the quality of evidence (GRADE). For the non-Cochrane reviews, only just over half had assessed the quality of evidence.

**Conclusion:**

Systematic reviews of interventions frequently rate randomised trials as high or unclear ROB for sequence generation. In general, Cochrane reviews were more transparent than non-Cochrane reviews in ROB and quality of evidence assessment. The scientific community should more strongly promote consistent ROB assessment for sequence generation to minimise selection bias and support transparent quality of evidence assessment. Consistency ensures that appropriate conclusions are drawn from the data.

## Background

Systematic reviews are a summary of the best available evidence and, as a result, may shape policy and help inform healthcare decisions [1]. Randomised trials are regarded as the optimal design to evaluate the effectiveness of healthcare interventions, and thus systematic reviews of intervention trials are an indisputable asset to clinical decision-making and evidence-based practice [2].

Generation of a sequence of random numbers is an essential component of randomisation and determines which groups trial participants are allocated to and, when used alongside effective allocation concealment, minimises the risk of selection bias [2]. Effective randomisation minimises bias in effect estimates, whereas inadequate randomisation may exaggerate treatment effects [3–5]. The Cochrane risk of bias (ROB) tool for interventions includes seven domains on which biases within trials are assessed, i.e. (1) sequence generation, (2) allocation concealment, (3) blinding of participants and personnel, (4) blinding of outcome assessment, (5) incomplete outcome data, (6) selective reporting and (7) any other [2]. If a systematic review of randomised trials states explicitly that non-randomised trials are excluded, then one would expect that sequence generation was truly random and therefore at low ROB for sequence generation. However, we have observed that some Cochrane and non-Cochrane reviews which a priori exclude non-randomised trials still report sequence generation as high or unclear ROB for some trials. Whereas a judgement of unclear ROB may be due to poor reporting in primary studies, it creates uncertainty whether non-randomised trials were truly excluded from the review. A judgement of high ROB for sequence generation suggests that non-randomised studies have likely been included.

Cochrane reviews and some non-Cochrane reviews authors now also judge the quality of evidence for the primary and sometimes secondary outcomes using the Grading of Recommendations Assessment, Development and Evaluation system (GRADE) (http://www.gradeworkinggroup.org/) or an equivalent assessment tool. A GRADE profile is performed for each outcome and includes an assessment of study limitations and subsequent downgrading if appropriate. If there is high ROB in trials examining a particular outcome, the GRADE quality of evidence would be downgraded due to study limitations. Thus, inconsistencies in ROB assessment can potentially impact the quality of evidence judgement which in turn could affect subsequent recommendations for practice and research.

In this paper, we examine the consistency in the ROB assessment of the sequence generation domain of the Cochrane ROB tool for randomised trials in Cochrane and non-Cochrane reviews that use this tool, and, if conducted, we further explored the authors’ judgement of the quality of evidence for the related outcomes.

The objectives of this study were:

1. To determine the proportion of reviews that state they include randomised trials only and judge trial(s) as high ROB or unclear ROB for sequence generation (and reason why, if given)
2. To examine if included reviews conducted a quality of evidence assessment for the primary outcomes
3. To describe if review authors downgraded the quality of evidence for study limitations in the presence of studies rated as high/unclear ROB for sequence generation, including an examination of the reported justification
4. To compare Cochrane and non-Cochrane reviews in relation to objectives 1 and 3

## Methods

A descriptive cross-sectional survey of the ROB assessment domain of sequence generation in Cochrane and non-Cochrane reviews of randomised trials. No ethical approval was required since this study used data already in the public domain.

### Data collection and extraction

Cochrane reviews of intervention studies published in the first quarter of 2017 were identified and retrieved from the Cochrane Database of Systematic Reviews. In addition, we manually searched for and included systematic reviews published between April 2016 and March 2017 in ten general medical journals with the highest impact factors (IF) based on the Thomson Reuters 2015 ranking. These were New England Journal of Medicine (IF 59.558); Lancet (IF 44.002); JAMA (IF 37.684); BMJ (IF 19.697); Annals of Internal Medicine (IF 16.593); JAMA Internal Medicine (IF 14.000); PLOS Medicine (IF 13.585); BMC Medicine (IF 8.005); Journal of Internal Medicine (IF 7.803); and the Canadian Journal of Medicine (IF 6.724).

Only systematic reviews of randomised trials were included. Overviews of reviews, non-intervention reviews, intervention reviews of non-randomised trials and narrative reviews were excluded. Data were extracted using a purposefully designed data extraction form. Data were extracted on (1) Cochrane group (if applicable), journal (if applicable) and country of lead author; (2) scope (study designs included); (3) the number of included trials; (4) the number and percentage of randomised trials judged as high, low and unclear ROB for the sequence generation domain and the accompanying justification; (4) whether sensitivity analyses were conducted and, if so, the criteria used; and (5) the GRADE quality of evidence rating for all primary outcomes and whether the authors downgraded the quality, including the justification given by the review authors.

### Data analysis

We examined the sequence generation domain of the Cochrane ROB tool using descriptive statistics, including the proportions of randomised trials that were rated as high, low or unclear ROB for this domain. For reviews that rated any randomised trials as high ROB for sequence generation, the justification was examined and compared to guidance provided in the Cochrane handbook [2]. Any discrepancies were reported descriptively. We excluded non-Cochrane reviews from the analysis that used a tool to assess ROB other than the Cochrane ROB tool or that did not examine ROB to allow for appropriate comparison with Cochrane reviews.

For reviews that had rated any randomised trials as having high or unclear ROB for sequence generation, we examined the proportion that downgraded the quality of evidence (GRADE) for study limitations for all primary outcomes that included these high/unclear ROB studies. If the review authors had downgraded for study limitations, the reported justification was examined. We also examined the proportion of reviews that conducted sensitivity analysis by ROB.

We carried out the above analyses on (1) all reviews, (2) Cochrane reviews only and (3) non-Cochrane review only.

## Results

### Search results

**Fig. 1:**
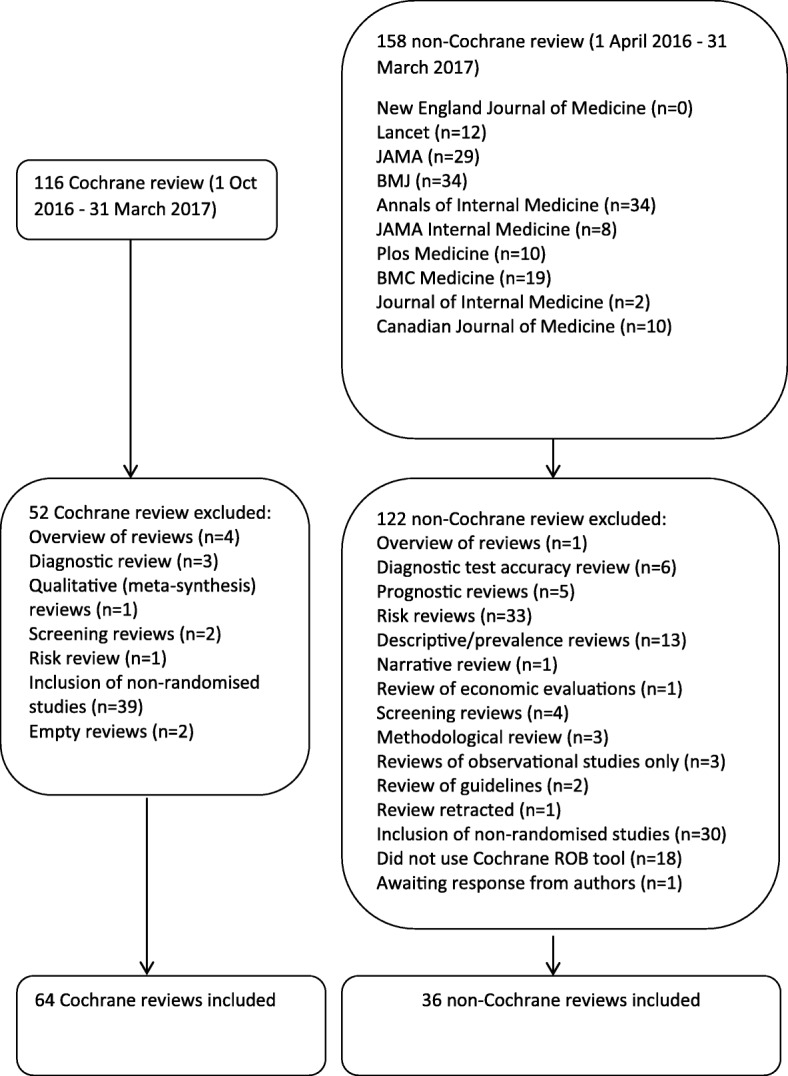
Search and selection flowchart

We identified 116 Cochrane reviews published between 1 October 2016 and 31 March 2017, of which 64 reviews were included in this study. We excluded 4 overviews of reviews, 3 diagnostic test accuracy reviews, 1 qualitative review/meta-synthesis, 2 screening reviews and 1 risk review. Thirty-nine reviews included non-randomised trials and were also excluded. Two Cochrane reviews were ‘empty’ reviews with no included studies. We identified 158 non-Cochrane reviews, of which 36 reviews had used the Cochrane ROB tool and were included in the final analysis. One review included individual patient data [6]. One of the included reviews did not report sufficient data; we contacted the authors but did not receive a response [7]. The search results and reasons for exclusion are presented in Fig. 1.

### Risk of bias of sequence generation for randomised trials

**Fig. 2:**
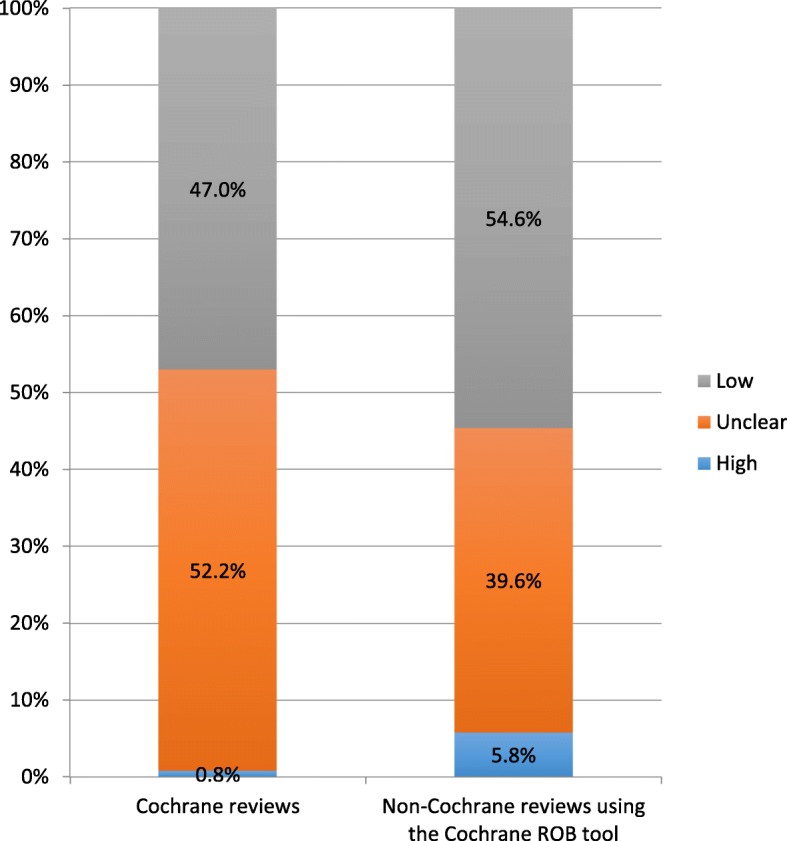
Risk of bias for sequence generation

The proportions of randomised trials rated as high, unclear and low ROB across all reviews are presented in Table 1 and Fig. 2. Fewer randomised trials were rated as having high ROB for sequence generation in Cochrane reviews (0.8%; *n* = 8) than in non-Cochrane reviews (5.8%; *n* = 80). The Cochrane review authors’ justifications for rating these eight randomised trials (across five reviews) as high ROB for sequence generation are presented in Table 2. All five reviews reported why the studies involved were rated as high ROB for sequence generation. However, when examining the reasons given, one review had rated a study as high ROB for sequence generation, but the justification said there was a lack of information reported, which would have been more appropriately rated as ‘unclear’ ROB [8]. The justification of one study [9] relates more to ROB related to allocation concealment than sequence generation.

**Table 1:** Risk of bias judgement for sequence generation of randomised trials in Cochrane and non-Cochrane reviews

Sequence generation ROB judgement of randomised trials	High ROB	Low ROB	Unclear ROB
Cochrane reviews^a^	8 (0.8%)	462 (47.0%)	514 (52.2%)
Non-Cochrane review using Cochrane ROB tool^b^	80 (5.8%)	751 (54.6%)	545 (39.6%)

^a^Sixty-four reviews, including 984 randomised trials

^b^Thirty-six reviews, including 1376 studies; one review is awaiting classification and contains an additional 35 randomised trials

**Table 2:** Reasons for rating randomised trials as high ROB for sequence generation in Cochrane reviews

Review	Number of randomised trials rated as high ROB for sequence generation (% of total included randomised trials)	Justification for high ROB for sequence generation (as stated by the review authors)
Lund et al. [13]	3 (75%)	Altinli 2007: “Participants were randomised into 2 groups, according to the day the participant was first seen in the clinic (odd and even days).” Sozen 2011a: “The participants were randomised into 2 groups—drained and fibrin sealant—according to the admission protocol number. Details of this protocol number unclear.” Sozen 2011b: “The participants were randomised into two groups, drained and non-drained, according to the admission protocol number. Nature of this protocol number unclear.”
Cheng et al. [9]	1 (16.7%)	Randomisation may have not been executed properly as there was a large difference in the number of participants in each arm; the acupuncture arm had 25/109 (40%) more participants than the control group. A random number table was used to generate sequence. Odd numbers were allocated to treatment group, even numbers were allocated to control group.
Chauhan et al. [14]	1 (2.7%)	Participants were randomised to 2 groups according to their order of presentation at the outpatient clinic.
McCaughan et al. [15]	1 (16.7%)	The randomisation protocol was compromised by selecting patients serially as they registered.
Menting et al. [8]	2 (6.9%)	Czibik-stable 2008 and Czibik-unstable 2008: “Randomisation not reported”

Eighty randomised trials across 11 non-Cochrane reviews were rated as high ROB for sequence generation. The reasons for judging these studies as high ROB for sequence generation are provided in Table 3. Ten of the 11 reviews did not report why the studies involved were rated high ROB for sequence generation. Around half of randomised trials in both Cochrane (52.2%; *n* = 514) and non-Cochrane (39.6%; *n* = 545) were rated as unclear ROB for sequence generation.

**Table 3:** Reasons for rating randomised trials as high ROB for sequence generation in non-Cochrane reviews

Review	Number of randomised trials rated as high ROB for sequence generation (% of total included randomised trials)	Reason for high ROB for sequence generation (as stated by the review authors)
Faruque et al. [16]	32 (28.8%)	(Not reported by review authors)
Hollingsworth et al. [17]	1 (1.8%)	(Not reported by review authors)
Cipriani et al. [18]	1 (2.9%)	(Not reported by review authors)
Collister et al. [19]	1 (5.9%)	(Not reported by review authors)
Eng et al. [20]	15 (51.7%)	(Not reported by review authors)
Franco et al. [21]	4 (6.5%)	(Not reported by review authors)
Hazlewood et al. [22]	8 (5.1%)	(Not reported by review authors)
Khera et al. [23]	1 (3.6%)	(Not reported by review authors)
Subramaniam et al. [24]	9 (10.5%)	(Not reported by review authors)
Schandelmaier et al. [25]	2 (7.7%)	Leung 2004: “Quasi randomised based on sequence of admission, Urita 2013: Used odd even system for treatment allocation”
Sukkar et al. [10]	6 (7.1%)	(Not reported by review authors)

### Quality of evidence assessment

All Cochrane reviews used the GRADE approach to examine the quality of evidence for each outcome. For the Cochrane reviews that included only randomised trials in their scope, 52.6% (*n* = 30) of reviews that had rated one or more randomised trials as high or unclear ROB for sequence generation had downgraded the quality of evidence for the corresponding primary outcomes (Table 4). Only two Cochrane reviews clearly reported which ROB domains (e.g. random sequence generation) contributed to the downgrading and provided a detailed statement of the number/size of studies that contributed to their judgement [9, 10]. Moreover, 17 (29.8%) of the reviews had conducted sensitivity analysis by ROB and 22 (38.6%) had planned sensitivity analysis by ROB but were unable to carry out the analysis due to insufficient data or because all included studies were of high ROB.

**Table 4:** Quality of evidence and sensitivity analysis in Cochrane reviews

Rated any randomised trials as high/unclear ROB for sequence generation	Downgraded for study limitations (additional reasons not specified)	Downgraded for study limitations (selection bias)	Sensitivity analysis based on ROB (conducted)	Sensitivity analysis based on ROB (planned; not able to conduct)
Cochrane reviews (*n* = 57)^a^	23 (40.4%)	7 (12.3%)	17 (29.8%)	22 (38.6%)

^a^Three of the 57 (5.3%) reviews did not downgrade at all and rated quality of evidence as high; 23 of the 57 (40.4%) reviews only downgraded for factors other than selection bias (other study limitations or other domains of the Cochrane ROB tool); 1 (1.8%) review did not report why they had downgraded the quality of evidence

For the non-Cochrane reviews, 20.8% (*n* = 5) of reviews that had rated at least one randomised trial as high or unclear ROB for sequence generation had downgraded the quality of evidence for the corresponding outcomes (Table 5). Only one non-Cochrane review that had rated at least one randomised trial as high or unclear ROB for sequence generation had conducted sensitivity analysis by ROB [11].

**Table 5:** Quality of evidence and sensitivity analysis in non-Cochrane reviews

Rated any randomised trials as high/unclear ROB for sequence generation)	Quality assessment using GRADE		Quality assessment using a tool other than GRADE		Conducted sensitivity analysis based on ROB
	Downgraded for study limitations (not specified)	Downgraded for study limitations (selection bias)	Downgraded for study limitations (not specified)	Downgraded for study limitations (selection bias)	
Non-Cochrane reviews (*n* = 24)^a^	1 (4.2%)	2 (8.3%)	2 (8.3%)	0	1 (4.2%)

^a^Eleven of the 24 (45.8%) reviews did not conduct any assessment of quality of evidence; 1 of the 24 (4.2%) reviews did not downgrade at all and rated quality of evidence as high; 7 of the 24 (29.2%) reviews only downgraded for factors other than selection bias despite risk of bias being part of the assessment tool used

## Discussion

Cochrane reviews judged randomised trials as high risk of bias for sequence generation less frequently compared to non-Cochrane reviews. More importantly, the reasons for this judgement were always reported, while only one of the ten non-Cochrane reviews reported the reason for rating some randomised trials as high ROB for sequence generation in their published material. This is likely to be attributed to the highly structured approach of conducting and reporting Cochrane reviews and the word count of other journals might limit authors’ ability to provide more detail. Nevertheless, it is beneficial to the research community to report this information in supplementary documents.

Approximately half of reviews, both Cochrane and non-Cochrane, judged at least one randomised trial as having unclear ROB for sequence generation. A lack of reporting in primary studies does not allow review authors to assess whether randomisation was adequate. Poor reporting is likely to be a greater issue in older studies, prior to the publication of reporting guidelines, although we did not stratify studies by year of publication for the purpose of this study. We only included non-Cochrane reviews that used the Cochrane ROB tool for comparison; subsequently, the full extent of ROB of sequence generation for the remaining body of evidence that used another tool or did not examine ROB was not examined.

Adequate randomisation together with allocation concealment minimise selection bias [3], which, if present, should be taken into account in the conclusions of the reviews. The assessment of the quality of evidence for individual outcomes can facilitate this process in a transparent way by appropriately downgrading the quality of evidence on which conclusions are based. Our findings show that slightly more than half of Cochrane reviews that included randomised trials of unclear or high ROB for sequence generation had downgraded the quality of evidence. This proportion was lower in non-Cochrane reviews; approximately one fifth of non-Cochrane reviews had adjusted the quality of evidence. However, in interpreting these findings, it is important to take into account that the decision to downgrade the quality of evidence is based on multiple factors, and including studies with high/unclear ROB for sequence generation does not necessarily indicate that downgrading is warranted. Whether or not the reviews should or should not have downgraded for study limitations is therefore uncertain, particularly since this is a judgement and the GRADE guidance to assess the quality of evidence is not rigid. When we examined the justification for downgrading, nearly all reviews only stated that they downgraded for study limitations due to (very) serious ROB, and, even if the specific types of bias (e.g. selection bias) that contributed to the decision to downgrade were reported in some reviews, only two Cochrane reviews provided further details with regards to the number/size of included studies that had biases. This suggests that transparency of the decision to downgrade is often lacking, even in Cochrane reviews.

The findings of our study raise questions about the conduct of systematic reviews, more specifically the ROB and quality of evidence assessment. We hope our findings will generate a debate concerning some key emerging issues for systematic review methodology. First, whether a study should be considered a randomised trial just because the study authors identified their study as a randomised trial, or whether this should be based on an assessment of the reporting of the methodological components required to classify as a randomised trial. This has implications for study selection in systematic reviews that include only randomised trials. Secondly, this study underscores that assessing quality of evidence for the outcome of interest could lead to better judgement of review findings and more accurately inform conclusions. All Cochrane reviews had conducted a quality of evidence assessment, reflecting the recent efforts of Cochrane to include this across all reviews. Regardless of the decision of whether to downgrade or not, this decision should be transparent from the onset of the review, ideally in the protocol phase, and the justification should be reported. Such was the case in all Cochrane reviews in line with recommendations from the GRADE Working Group [12]; however, the justifications provided often lack detail to clearly follow the reasoning of the judgement. In contrast, only 54% (*n* = 13) of non-Cochrane reviews had assessed the quality of evidence. For reviews that rated randomised trials as high/unclear ROB for sequence generation, it might be difficult to ascertain how this did or did not affect the conclusions of a review if they did not conduct a formal quality of evidence assessment.

This study examined only the sequence generation domain of the ROB assessment. Selection bias can be introduced by an inadequate sequence, but can also result from inadequate or absence of allocation concealment or blinding. Further research could look at these domains in combination. For the findings regarding the quality of evidence, it was not possible to assess the appropriateness, or not, of downgrading the quality of evidence because of the inclusion of studies of high ROB for sequence generation, since this decision is based on multiple factors, not all examined in this study and often not reported in reviews.

## Conclusions

Cochrane and non-Cochrane systematic reviews of interventions frequently rate randomised trials as high or unclear ROB for sequence generation. Just under half of non-Cochrane reviews did not conduct a quality of evidence assessment, but all Cochrane reviews did. It is important for the scientific community to increase efforts promoting consistency and transparency in the ROB and quality of evidence assessment in systematic reviews to minimise bias in the review process. A structured approach to conducting systematic reviews (such as in Cochrane reviews) and to assessing the quality of evidence may provide more transparency in the reviews’ conclusions, which is critical given that systematic reviews are frequently used to guide clinical practice. Our findings emphasise the importance of good reporting in primary studies to facilitate the review process.

## Abbreviations

ACCF/AHA: American College of Cardiology Foundation/American Heart Association
EPOC: Effective Practice and Organisation of Care
GRADE: Grading of Recommendations Assessment, Development and Evaluation system
IF: Impact factor
MERSQI: Medical Education Research Study Quality Instrument
ROB: Risk of bias
USPSTF: US Preventative Services Task Force

## References

[1] Lavis J, Davies H, Oxman A, Denis JL, Golden-Biddle K, Ferlie E (2005). Towards systematic reviews that inform health care management and policy-making. J Health Serv Res Policy.

[2] Higgins JGS. Cochrane handbook for systematic reviews of interventions version 5.1.0 [updated March 2011]: Cochrane Collaboration; 2011. http://handbook-5-1.cochrane.org/.

[3] Odgaard-Jensen J, Vist GE, Timmer A, Kunz R, Akl EA, Schunemann H, Briel M, Nordmann AJ, Pregno S, Oxman AD (2011). Randomisation to protect against selection bias in healthcare trials. Cochrane Database Syst Rev.

[4] Als-Nielsen B, Gluud LL, C G: Methodological quality and treatment effects in randomized trials: a review of six empirical studies. 12th Cochrane Colloquium, Ottawa: Cochrane collaboration. 2004. https://abstracts.cochrane.org/2004-ottawa/methodological-quality-and-treatment-effects-randomised-trials-review-six-empirical.

[5] Armijo-Olivo S, Saltaji H, da Costa BR, Fuentes J, Ha C, Cummings GG (2015). What is the influence of randomisation sequence generation and allocation concealment on treatment effects of physical therapy trials? A meta-epidemiological study. BMJ Open.

[6] Kotecha D, Manzano L, Krum H, Rosano G, Holmes J, Altman DG, Collins PD, Packer M, Wikstrand J, Coats AJS (2016). Effect of age and sex on efficacy and tolerability of β blockers in patients with heart failure with reduced ejection fraction: individual patient data meta-analysis. BMJ.

[7] Wilt TJ, MacDonald R, Brasure M (2016). Pharmacologic treatment of insomnia disorder: an evidence report for a clinical practice guideline by the american college of physicians. Ann Intern Med.

[8] Menting TP, Wever KE, Ozdemir-van Brunschot DM, Van der Vliet DJ, Rovers MM, Warle MC (2017). Ischaemic preconditioning for the reduction of renal ischaemia reperfusion injury. Cochrane Database Syst Rev.

[9] Cheng K, Law A, Guo M, Wieland LS, Shen X, Lao L (2017). Acupuncture for acute hordeolum. Cochrane Database Syst Rev.

[10] Sukkar L, Hong D, Wong MG, Badve SV, Rogers K, Perkovic V, Walsh M, Yu X, Hillis GS, Gallagher M (2016). Effects of ischaemic conditioning on major clinical outcomes in people undergoing invasive procedures: systematic review and meta-analysis. BMJ.

[11] Kavalieratos D, Corbelli J, Zhang D (2016). Association between palliative care and patient and caregiver outcomes: a systematic review and meta-analysis. JAMA.

[12] Guyatt GH, Oxman AD, Vist G, Kunz R, Brozek J, Alonso-Coello P, Montori V, Akl EA, Djulbegovic B, Falck-Ytter Y, Norris SL, Williams JW Jr, Atkins D, Meerpohl J, Schünemann HJ. GRADE guidelines: 4. Rating the quality of evidence--study limitations (risk of bias). J Clin Epidemiol. 2011;64(4):407-15. 10.1016/j.jclinepi.2010.07.017 21247734

[13] Lund J, Tou S, Doleman B, Williams JP (2017). Fibrin glue for pilonidal sinus disease. Cochrane Database Syst Rev.

[14] Chauhan BF, Jeyaraman MM, Singh Mann A, Lys J, Abou-Setta AM, Zarychanski R, Ducharme FM (2017). Addition of anti-leukotriene agents to inhaled corticosteroids for adults and adolescents with persistent asthma. Cochrane Database Syst Rev.

[15] McCaughan E, Parahoo K, Hueter I, Northouse L, Bradbury I (2017). Online support groups for women with breast cancer. Cochrane Database Syst Rev.

[16] Faruque LI, Wiebe N, Ehteshami-Afshar A, Liu Y, Dianati-Maleki N, Hemmelgarn BR, Manns BJ, Tonelli M (2017). Effect of telemedicine on glycated hemoglobin in diabetes: a systematic review and meta-analysis of randomized trials. CMAJ.

[17] Hollingsworth JM, Canales BK, Rogers MAM, Sukumar S, Yan P, Kuntz GM, Dahm P (2016). Alpha blockers for treatment of ureteric stones: systematic review and meta-analysis. BMJ.

[18] Cipriani A, Zhou X, Del Giovane C, Hetrick SE, Qin B, Whittington C, Coghill D, Zhang Y, Hazell P, Leucht S (2016). Comparative efficacy and tolerability of antidepressants for major depressive disorder in children and adolescents: a network meta-analysis. Lancet.

[19] Collister D, Komenda P, Hiebert B (2016). The effect of erythropoietin-stimulating agents on health-related quality of life in anemia of chronic kidney disease: a systematic review and meta-analysis. Ann Intern Med.

[20] Eng J, Wilson RF, Subramaniam RM (2016). Comparative effect of contrast media type on the incidence of contrast-induced nephropathy: a systematic review and meta-analysis. Ann Intern Med.

[21] Franco OH, Chowdhury R, Troup J (2016). Use of plant-based therapies and menopausal symptoms: a systematic review and meta-analysis. JAMA.

[22] Hazlewood GS, Barnabe C, Tomlinson G, Marshall D, Devoe D, Bombardier C (2016). Methotrexate monotherapy and methotrexate combination therapy with traditional and biologic disease modifying antirheumatic drugs for rheumatoid arthritis: abridged Cochrane systematic review and network meta-analysis. BMJ.

[23] Khera R, Murad M, Chandar AK (2016). Association of pharmacological treatments for obesity with weight loss and adverse events: a systematic review and meta-analysis. JAMA.

[24] Subramaniam RM, Suarez-Cuervo C, Wilson RF (2016). Effectiveness of prevention strategies for contrast-induced nephropathy: a systematic review and meta-analysis. Ann Intern Med.

[25] Schandelmaier S, Kaushal A, Lytvyn L, Heels-Ansdell D, Siemieniuk RAC, Agoritsas T, Guyatt GH, Vandvik PO, Couban R, Mollon B (2017). Low intensity pulsed ultrasound for bone healing: systematic review of randomized controlled trials. BMJ.

